# The Adulteration of Food

**Published:** 1908-12-05

**Authors:** 


### SPECIAL ARTICLE.

### THE ADULTERATION OF FOOD.

Fraud and adulteration are ugly words, and their ugliness is by no means diminished by their application to food and food products. Modern research has told us that the mind and the fancies of it have no insignificant influence upon the processes of digestion, and the fear of what we eat being adulterated may not only oppress our minds, but may equally hamper our digestions. That some hundreds of merchants, and some tens of chemists and food experts of several nations, should have spent a week at Geneva discussing both the existence and the possibility of the prevention of fraud in relation to food products is certainly evidence both of the existence of fraud in this branch of industry, and also of the extensiveness of the food areas implicated. A weight will, however, at once be lifted off the mind of the ordinary consumer when he learns that by far the greater part of the frauds discussed at Geneva do not essentially affect him.

The terms fraud and adulteration are by no means synonymous, for while adulteration is always fraud, fraud is not always adulteration. Fraud, at any rate so far as this last Congress was concerned, may exist and be practised by one section of the trade as against another; one section of the trade may produce an article which may infringe a description applied to it by another, and may in that sense be fraudulent, but not, so far as the consumer is concerned, adulterated. This kind of fraud does not interest us for the moment, and is dealt with in this country by the Merchandise Marks Act. The second variety of fraud is adulteration, and consists in supplying an article which may be said, from the actual point of view of the consumer, to masquerade under the name applied to it, and not to be of the quality and substance demanded. Adulteration clearly affects the consumer, and it is of general interest to note how the Congress at Geneva dealt with it. The method adopted was what may be termed the method of definitions; it defined pure food, and thus by exclusion assumed that it defined impure food. Since at least one reason, so far as France was concerned, for this Congress was the acquiring of information from the trade with regard to the working of the 1905 pure food law, this method is of interest.

The application of modern technology to the preparation of food products, a necessary result of the development of applied science, has given rise to many complications, and to the reconsideration of the whole question of adulteration. Different countries have endeavoured to meet it in different ways, and for the most part by new so-called pure food laws. Unfortunately in these laws and considerations two issues are often intermingled and confused—namely, the good of the consumer as an individual, and the general good of the trade or the nation, of which the consumer is a part. In other words, regulations intended essentially to protect certain industries or certain classes of industry, instead of having their object openly avowed, are covered by the euphonious shibboleth of the public health. Reasons purely economic are confounded with those purely hygienic. It is the object, and the perfectly justifiable object, of certain countries to restrict the importation of food; it is equally the object of certain members of the population to restrict the transportation of food from one part of a given country to another. This is best done honestly and openly, and not by designating home or locally produced food pure and imported or transported food impure.

English law, which naturally gives expression to the necessity for both the importation and the transportation of food, has very rarely descended to definitions. It has formulated and maintains two main principles with regard to food, both absolutely in the interest of the consumer—namely, that food must never, under any conditions, contain substances injurious to health, and that the bulk or weight of food must not be fraudulently added to, that is, increased with the object of diminishing the actual amount of food substance present.

The foods most especially concerned by the above considerations are butter, milk, cream, and cheese, products for the most part natural, but nevertheless necessarily undergoing some manipulation before reaching the consumer. It may here be mentioned that this actual word manipulation was freely admitted by the definers of pure food, and the manipulations to which pure food may be submitted without presumably becoming impure are to be considered and formulated upon a subsequent occasion. This really must follow because the term pure, as applied to food, although an appetising, is not a particularly happy one. Pure, in the minds of most of us, implies the idea at any rate of a certain degree of uniformity. Pure milk should be milk the chemical composition of which should only vary within very narrow limits, and the same would apply to butter; unfortunately this is only partially true, and hence the attribute of pure must demand the formulation of a standard of purity, and some individual members of any given class of food can only be rendered pure by being artificially brought up or lowered down to this standard, or, in other words, by being first rendered impure. If, indeed, we take another view of the word pure as applied to food, and regard it as synonymous with natural, we are involved in worse difficulties, for under these circumstances a buyer, without he is an expert, would probably pay the same price for rich milk as for poor, both of which are in the real sense natural—*i.e.* pure. When we come to consider a less natural product like butter, this terminology involves us in still greater difficulties. The English law has defined butter not pure butter, but commercial butter, and it is interesting to note, as Dr. Tunnicliffe pointed out at the Congress, that the only definition of any food stuff ascribed to England—namely, that of butter—was quite incorrect. The English definition of butter defines the materials from which it shall be made, limits the quantity of water which it shall contain, and admits the addition of salt and a preservative not injurious to health. The tentative definition of pure butter adopted at the Congress defines the materials from which it shall be made, precludes the addition of salt or preservative, and does not limit the amount of water. The price of butter in Paris is, roughly speaking, 1s. 8d. a pound; in London 1s. 2d. The object of France, as clearly defined by M. Ruau, the Minister of Agriculture, at the Congress, is to maintain a policy of protection, and the extent to which this definition of butter conduces to this end will be appreciated by those who know the ins and outs of the French butter trade.

A by-issue of considerable importance in this connection, and only touched upon by the Congress, is the question of the use of preservatives in food stuffs of all kinds, especially in those of a perishable nature. Chief among preservatives is boracic acid, the preservative generally used in preserved meats, butter, and sausages. This substance, provided it is added to originally sound food, certainly facilitates both the transportation of food from one part of a country to another, and also the importation of food from abroad, and also the keeping of food in a sound condition in the domestic larder, the hygienic condition of which often in crowded London leaves a good deal to be desired. In fact, this substance possesses the preservative power of salt without its salt taste, and it in this sense facilitates the supply of what the public demand—namely, a perishable article of food which is fresh, *i.e.* not salt, and yet will keep. Whether all this good is to be obtained without any evil, this is not the place to discuss. The same applies to other preservative agents when used in other foods. The point to be emphasised here is that the issue considered should be a clear one, and that those countries or communities who wish to hinder, or, at any rate, not to further, the importation or transportation of food should not condemn any agency tending to help it for other reasons than the actual one in their minds. In other words, protection should not masquerade under the cloak of hygiene.

The definitions of the Congress, so far as more complicated food products were concerned—that is, products in which there is less of nature and more of art—were essentially imbued with the same "motif": certainly they were not all framed in the direct hygienic interest of the consumer. They are, however, all interesting as showing how complicated are the interests involved in the manipulations and manufacture of food, and for this reason how insuperably difficult is the formulation of regulations likely to be in the real sense international.

